# Estrogen-related receptor alpha (ERRα) promotes gastrointestinal stromal tumor progression and epithelial-mesenchymal transition through Wnt/β-catenin

**DOI:** 10.21203/rs.3.rs-9930164/v1

**Published:** 2026-06-24

**Authors:** Ronald DeMatteo, Taylor Hartlein, Shan Zeng, Ferdinand Rossi, Montana Morris, Jonathan Sussman, Michael Beckman, Iulia Barbur, Kevin Do, Juan Esteban Perez, Gabrielle Cole, Danielle Fortuna, Jake Mlakar, E. Petersson

**Affiliations:** Perelman School of Medicine, University of Pennsylvania; Perelman School of Medicine, University of Pennsylvania; Perelman School of Medicine, University of Pennsylvania; Perelman School of Medicine, University of Pennsylvania; Perelman School of Medicine, University of Pennsylvania; Perelman School of Medicine, University of Pennsylvania; Perelman School of Medicine, University of Pennsylvania; Perelman School of Medicine, University of Pennsylvania; Perelman School of Medicine, University of Pennsylvania; Perelman School of Medicine, University of Pennsylvania; Perelman School of Medicine, University of Pennsylvania; Perelman School of Medicine, University of Pennsylvania; Hospital of the University of Pennsylvania; Perelman School of Medicine, University of Pennsylvania

## Abstract

Gastrointestinal stromal tumor (GIST) is the most common human sarcoma and primarily driven by a gain-of-function mutation in either the KIT or PDGFRA receptor tyrosine kinase. Estrogen-related receptor alpha (ERRα, encoded by *ESRRA*) is an orphan nuclear receptor that shares structural homology and overlapping transcriptional targets with estrogen receptor alpha (ERα). We found that ERRα was highly expressed in human GIST cell lines and human GIST surgical specimens. Pharmacologic inhibition of ERRα using the inverse agonist XCT790 or siRNA-mediated knockdown of ERRα reduced viability, colony formation, and migration of human GIST cell lines. Furthermore, XCT790 suppressed the growth of established tumor xenografts. ERRα knockdown in GIST T1 cells reduced Hallmark Epithelial-Mesenchymal Transition (EMT) by gene set enrichment analysis of RNA-seq data and β-catenin was identified as a key differentially expressed gene. Lastly, ERRα co-immunoprecipitated with nuclear β-catenin and ERRα knockdown decreased levels of active nuclear β-catenin. Thus, ERRα promotes tumor progression and EMT in GIST, revealing a novel molecular pathway and potential therapeutic target.

## INTRODUCTION

Gastrointestinal stromal tumor (GIST) is the most common human mesenchymal tumor, arising from specialized pacemaker cells in the gut known as the interstitial cells of Cajal (1). GISTs most frequently originate in the stomach and small intestine, but can occur anywhere along the gastrointestinal tract (1). The molecular pathogenesis of GIST is characterized by gain-of-function mutations in receptor tyrosine kinases (RTKs), with approximately 80% of cases harboring mutations in *KIT* and an additional 10% in *PDGFRA* (2). These mutations result in constitutive RTK activation of downstream signaling pathways, including PI3K/AKT, MAPK/ERK, and STAT, culminating in uncontrolled cell proliferation and survival (2). Imatinib mesylate, the first line therapy, and other tyrosine kinase inhibitors (TKIs) reduce these oncogenic signaling cascades. Though imatinib has dramatically improved outcomes for advanced GIST, most patients eventually develop secondary resistance and subsequent TKIs generally have short-lived efficacy (2). Resistance typically results from a secondary KIT mutation that reduces TKI binding, but there are likely other non-genetic mechanisms (3). Importantly, many of these non-genetic adaptations are thought to emerge early during TKI treatment, prior to the acquisition of secondary kinase mutations, suggesting that preventing or delaying this initial adaptive reprogramming may be therapeutically critical.

Estrogen-related receptor alpha (ERRα) is encoded by the *ESRRA* gene and is an orphan nuclear hormone receptor. Despite its structural homology to estrogen receptor-alpha (ERα), ERRα is not activated by hormone binding. Instead, it is constitutively expressed in most tissues and depends on co-factor binding for transcriptional activity (4). ERα and ERRα both can bind estrogen response elements (EREs) and therefore share some transcriptional targets, but they largely maintain independent and distinct transcriptional programs (5). ERRα is a master regulator of metabolism, primarily in tissues expressing the co-activators PPARγ coactivator-1α and β (PGC-1α and PGC-1β), and controls differentiation of mesenchymal stem cells during development (6).

Epithelial-mesenchymal transition (EMT) is a cellular reprogramming process whereby cells lose epithelial characteristics, such as cell-cell adhesion and apical-basal polarity, and acquire mesenchymal properties that confer enhanced migratory and invasive capabilities (7). This process is mediated by the transcription factors Snai1/2, ZEB, and Twist1/2 and characterized by the gradual loss of E-cadherin and simultaneous upregulation of mesenchymal markers, such as N-cadherin, vimentin, fibronectin, and matrix metalloproteinases (MMPs). This is a reversible and dynamic process, as cells are able to regain an epithelial phenotype or exist in hybrid states with varying degrees of epithelial and mesenchymal characteristics (7). For mesenchymal tumors, like GIST, tumor cells already exist in a mesenchymal state, so it is less likely that sarcomas fully undergo EMT as epithelial tumors do. However, it is not uncommon for sarcomas to express some epithelial markers and exist in varying degrees of epithelial or mesenchymal differentiation. It has been proposed that some sarcomas reside in an intermediate EMT state and have the ability to fluctuate between a more mesenchymal-like or more epithelial-like state, which can ultimately influence aggressiveness and metastatic potential (8). For example, in osteosarcoma and synovial sarcoma, a shift towards the mesenchymal state promotes tumor invasion, migration, and metastatic spread (9, 10)

Here, we tested the hypothesis that ERRα drives GIST pathogenesis. Using a human GIST tissue microarray, pharmacologic and genetic ERRα inhibition in human GIST cell lines, whole-transcriptome RNA sequencing, and xenograft modeling, we identify ERRα as a previously uncharacterized driver of GIST progression that promotes tumor growth, EMT, and cell migration. We further demonstrate that ERRα regulates EMT at multiple upstream transcriptional nodes and engages the Wnt/β-catenin pathway through direct physical association with nuclear β-catenin. These findings establish the ERRα–β-catenin axis as a therapeutic vulnerability in GIST and suggest that ERRα inhibition may represent a strategy for increasing the effects of tyrosine kinase inhibitors and overcoming adaptive TKI resistance.

## MATERIALS AND METHODS

### Animals.

Age- and sex-matched *Kit*^*ΔV558/+*^ mice were maintained in a pathogen-free animal facility and used in accordance with an Institutional Animal Care and Use Committee (IACUC) approved protocol. Ovariectomy was performed on 5-week-old female *Kit*^*ΔV558/+*^ mice under inhalational Isoflurane via a single dorsal incision, sharp dissection of the lumbar fascia, and removal of the ovaries under direct visualization. Controls underwent sham operation. 5-week-old female *Kit*^*ΔV558/+*^ mice and 14-week-old male *Kit*^*ΔV558/+*^ mice were treated with daily tamoxifen gavage (100mg/kg) or vehicle for 3 weeks prior to tumor harvest. NOD SCID gamma (NSG) mice were bred in our institutional facility.

### Cell culture.

Human GIST-T1 (RRID:CVCL_4976) and HG129 cell lines have *KIT* exon 11 deletions and are imatinib-sensitive (11, 12). HG130 has exon 11 W557R + 13 V654A mutations and is imatinib and sunitinib-resistant (13). GIST cell lines were cultured in RPMI-1640 supplemented with L-glutamine and 25 mmol/L HEPES, 10% fetal bovine serum (FBS), and 1% penicillin and streptomycin. MCF7 (RRID: CVCL_0031) is an ER+/PR+ breast cancer cell line that expresses high levels of ERRα and was used as a positive control. MCF7 cells were maintained in DMEM supplemented with 4.5g/L D-glucose and L-glutamine, 10% FBS, 1% non-essential amino acids, and antibiotics. All cell lines were Mycoplasma tested and underwent authentication within the last year. Cells were plated, treated at 24h, and harvested 48–72h later.

### GIST xenograft models.

10^6^ GIST T1 or HG130 cells in 200 μl of a 1:1 PBS/Matrigel solution were inoculated into the flanks of NSG. Tumors were measured weekly and treatment started when mean tumor volume exceeded 100mm^3^. Mice were randomized to vehicle, imatinib, or XCT790. Imatinib (600 mg/L in drinking water) and XCT790 (5–10 mg/kg by daily gavage) had different routes of administration thus two controls were utilized - normal drinking water and saline gavage, respectively.

### Patient tumors.

Human GIST surgical specimens were obtained in accordance with an Institutional Review Board approved protocol. Patients provided preoperative written informed consent and studies were conducted in accordance with the Declaration of Helsinki. Patient information was de-identified and encrypted in compliance with the Health Insurance Portability and Accountability Act.

### Drug formulation and administration.

Tamoxifen was dissolved in 5% EtOH and corn oil and 100 mg/kg was administered by daily oral gavage. Fulvestrant was dissolved in corn oil and 0.5 mg/kg was administered daily s.c. For in vitro experiments, XCT790 was reconstituted in DMSO and diluted in maintenance media at various concentrations with an equivalent volume of DMSO used for control. For xenograft experiments, XCT790 (5–10 mg/kg) was reconstituted in DMSO and diluted in 40% PEG300 and 5% Tween80 and was administered as a daily gavage. Imatinib was kindly provided by Novartis and administered in the drinking water at 600 mg/L in xenograft experiments. In vitro, 50 nM imatinib was diluted in growth media and given alone or combined with XCT790 or non-target siRNA (RNA-seq). In rescue experiments after ERRα or non-target knockdown, cells were treated with 100 ng/ml of Wnt3a or Wnt5a to stimulate the canonical and non-canonical Wnt/β-catenin signaling pathways, respectively.

### Viability assay.

96-well plates were seeded with 10^4^ cells/well and treated with various concentrations of XCT790 or DMSO. There was a minimum of 10 replicates per treatment group. After 48–72h, viability was measured using Cell Counting Kit-8 per the manufacturer’s instructions, and values were normalized to control. XCT790 IC_50_ values were determined using nonlinear regression in Prism.

### Gene knockdown.

For ERRα knockdown, 6-well plates with 2×10^5^ T1 or HG129 cells/well were transfected at 50–60% confluence using 25 pmol/well ERRα siRNA or a non-target control siRNA and transduced with 7.5 μl/well Lipofectamine RNAiMAX in Opti-MEM media. 48h later, cells were harvested and processed for RNA sequencing, PCR, or western blot. For rescue experiments after knockdown, cells were stimulated with 100 ng/ml Wnt3a or Wnt5a for 4h prior to harvest. For viability assays, 96-well plates with 10^4^ cells/well were transfected with 1 pmol ERRα siRNA or non-target siRNA and cell viability was measured at 72h.

### Colony formation assay.

6-well plates were seeded with 10^3^ T1 or HG129 cells/well and treated with XCT790 or DMSO control after 24h until control wells had at least 50 cells/colony. Cells were then fixed in 4% paraformaldehyde for 10 minutes and stained with crystal violet. Colony intensity (normalized to control) and surface area were quantified using ImageJ software.

### Western Blotting and Immunoprecipitation.

Nuclear (NE) and cytoplasm (CE) extract lysates were made by using NE-PER Nuclear and Cytoplasm Protein Extraction Reagents. Western blot was run on 4–15% pre-cast gels as previously described (14). Membranes stained with anti-rabbit primary antibodies (**Supp. Table 1)** at 1:100 and developed in standard fashion. ImageJ used for densitometry quantification and normalized to loading control GAPDH or lamin β1. Immunoprecipitation (IP) was performed using the Dynabeads Protein A Immunoprecipitation Kit. ERRα (E1G1J), β-catenin (D10A8), or rabbit IgG (negative control) antibodies were added to NE MCF7 and HG129 lysates and immunoprecipitated and per manufacturer’s instructions. NE lysate of each cell line (positive internal control) was loaded onto gel followed by ERRα, β-catenin, and IgG immunoprecipitates and western blot performed in standard fashion.

### RNA sequencing.

RNA sequencing was performed on T1 cells after treatment with ERRα siRNA or non-target control, with 3 replicates in each group. Cells were harvested 48h later and gene knockdown was confirmed by PCR. Bulk RNA sequencing was performed on the Illumina HiSeq 2500 platform at the High-Throughput Sequencing Core of the Children’s Hospital of Philadelphia. Reads were aligned to the human genome (GRCh38) using STAR aligner with default parameters (15). Gene-count matrices were produced by featureCounts (16). To compare gene expression between samples, expression levels were normalized based on the “median of ratios” method using DESeq2 and differentially expressed genes (DEGs) were computed (17). DEGs were selected using the parameters log2FC ≤ −0.5 or ≥ 0.5 and padj (FDR) < 0.05. Log fold changes were shrunken using the *apeglm* method for purposes of visualization (18). For pathway analysis, the DESeq2 statistic was used as input to a pre-ranked gene set enrichment analysis (GSEA) using the fgsea package. Pathways were sourced from the Molecular Signatures Database Hallmark gene sets (19), the KEGG database (20), and the Reactome database (21), totaling 2,075 pathways, and FDR < 0.05 identified significantly enriched pathways. Up- and down-regulated DEGs were used to create a protein-protein interaction (PPI) network using the STRING database (22) and filtered using a minimum of 0.7 confidence interaction score. STRING network was then exported to Cytoscape (23) and cytoHubba plugin (24) was used to identify 7 hub genes ranked by maximal clique centrality (MCC). An EMT signature score was calculated with the Hallmark Epithelial-Mesenchymal Transition pathway using a robust Z-score aggregation approach (25). For each gene in the signature, expression values were normalized across samples by subtracting the median and dividing by a robust estimate of scale, defined as the arithmetic mean of the standard deviation and the median absolute deviation (MAD). To account for genes with zero variance, unscaled centered values were used if the robust scale estimate was zero or negative. The final signature score for each sample was computed as the mean of the robust z-scores for all genes in the signature set. Additionally, we supplemented our analysis of nuclear receptor expression using RNA-seq dataset GSE143547 (26). We also used RNA-seq data from 75 human GIST specimens (PRJNA521803) from our previously published work (27).

### Quantitative real-time PCR.

RNA was extracted from tumor tissues or cells using the RNAeasy Plus Mini kit and concentration was measured with NanoDrop 2000. 1μg RNA was reverse transcribed with the Taqman Reverse Transcription Kit and MultiscribeRT. PCR was performed with PCR Taqman probes (**Supp. Table 2**) and QuantStudio 5 PCR system. Data were analyzed using the 2^*− ΔΔCt*^ method as described in the manufacturer’s instructions and expressed as fold change over GAPDH control.

### Immunohistochemistry and Tissue Microarray.

Formalin-fixed and paraffin-embedded tumors were sectioned at 5μm thickness. Antigen retrieval was achieved with Dako Antigen Retrieval Solution. Slides were stained for ERRα, ERα, Ki67, Cyclin D1, and isotype control with antibodies validated for immunohistochemistry (**Supp. Table 1**). Slides were imaged using an Olympus BX5 brightfield microscope (20x) and DPController software. A tissue microarray was constructed with triplicate cores (4 μm sections) from 66 human GISTs normal testis, liver, and kidney controls. The primary antibody was polyclonal rabbit anti-ERRα (1:100). Staining was done on a Leica Bond-IIITM instrument using the Bond Polymer Refine Detection System. Heat-induced epitope retrieval was done for 20 minutes with ER2 solution. QuPath software was used to quantify percent staining defined as (positive staining cells/total cells) × 100, which were averaged across triplicate cores per patient.

### Scratch assay.

Cell monolayers in 12-well plates were grown to 80–90% confluency. Linear scratches were made with a 20 μl pipette tip. Then, wells were washed with PBS and treated with XCT790 or DMSO control in 5% FBS to suppress proliferation interference. Images of each well were acquired initially and again after 48h and ImageJ was used to calculate the scratch area at each time point. Scratch closure percentage was calculated with the following formula: [(initial area – 48h area)/initial area] × 100.

### Statistical analysis.

GraphPad Prism and BioRender (https://BioRender.com) were used for statistical analysis and data visualization. Continuous data are expressed as mean ± SEM or median. All data were assessed for normality using the Shapiro-Wilk test. When data satisfied both normality and equal variance assumptions, Student’s t-test, one-way ANOVA with Tukey post-hoc test, one-way ANOVA with Dunnett’s multiple comparisons test, or two-way ANOVA with Bonferroni’s multiple comparisons test were used, as appropriate for the experimental design. When data met normality assumptions but violated homoscedasticity, Welch’s one-way ANOVA with Games-Howell post-hoc test was applied. For data that violated normality, Mann-Whitney U test or Kruskal-Wallis test with Dunn’s post-hoc correction was used. A p-value < 0.05 was considered statistically significant.

## RESULTS

### Inhibiting ERα suppresses tumor growth in a GIST mouse model.

**Figure 1:**
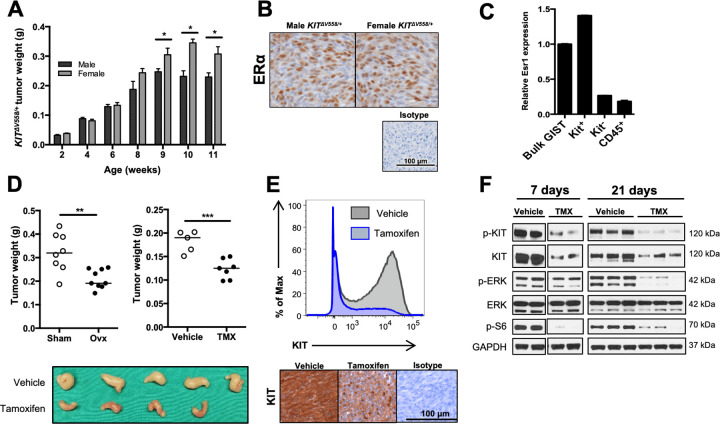
Estrogen receptor alpha (ERα) promotes GIST tumor growth in *KIT*^*ΔV558/+*^ mice. **A)** Tumor weights in male and female *Kit*^*ΔV558/+*^ mice at various ages (mean ± SEM). **B)** Representative ERα staining in *Kit*^*ΔV558/+*^ tumors of 6-week-old male and female mice (40x, 100 μM scale). **C)** Relative *Esr1* mRNA expression by qPCR in flow-sorted cell populations (Kit^+^, Kit^−^, and CD45^+^) of *Kit*^*ΔV558/+*^ tumors normalized to unsorted bulk tumors. **D)** Tumor weights following ovariectomy (Ovx; n=9) compared to sham surgery (n=8), or 3 weeks of daily oral tamoxifen (n=7) or vehicle (n=5) gavage in female *Kit*^*ΔV558/+*^ mice. Medians are indicated. **E)** KIT expression by flow cytometry and representative IHC (40x, 100 μM scale) in *Kit*^*ΔV558/+*^ tumors following tamoxifen or vehicle treatment for 3 weeks. **F)** Western blot analysis of *Kit*^*ΔV558/+*^ tumors after 7 or 21 days of tamoxifen treatment. Statistical significance assessed with unpaired Student’s t-test; *p<0.05, **p<0.01, ***p<0.001.

*Kit* ^*ΔV558/+*^ mice spontaneously develop a cecal GIST with 100% penetrance (28). We noticed that after 6 weeks of age (when sexual maturity occurs), females had larger tumors than age-matched males (Fig. 1A). ERα staining was seen in both female and male tumors (Fig. 1B). ERα mRNA expression was higher in isolated Kit^+^ cells compared to Kit^−^ cells and CD45^+^ immune cells (Fig. 1C). To determine if increased circulating estrogen was contributing to the larger tumors in females, ovariectomy was performed on 5-week-old female mice, which resulted in smaller tumors compared to sham operation (Fig. 1D). Alternatively, to inhibit stimulation of ERα by estrogen, female mice were treated with tamoxifen, which consistently reduced tumor size, Kit expression, and downstream Kit signaling (Fig. 1D–F). Treatment with fulvestrant, a selective ERα inhibitor, similarly suppressed tumor growth, confirming that tamoxifen’s anti-tumoral activity was mediated primarily through ERα blockade (**Supp.** Figure 1).

### Human GIST expresses high levels of ERRα but not ERα.

**Figure 2:**
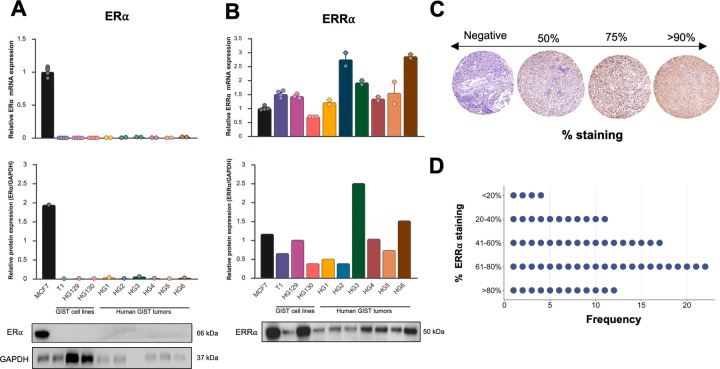
Estrogen-related receptor alpha (ERRα) is highly expressed in human GIST while ERα is absent. The human GIST cell lines (T1, HG129, HG130), human GIST tumor specimens (HG1–6), and the breast cancer cell line MCF7 were assessed. **A)** Relative ERα (*ESR1*) mRNA expression by qPCR (top) and protein levels by densitometry of western blot (bottom). **B)** Relative ERRα (*ESRRA*) mRNA expression by qPCR (top) and protein levels by densitometry of western blot (bottom). Note the GAPDH controls are the same as in panel A. **C)** ERRα immunohistochemistry of a primary human GIST tissue microarray (n=66) illustrating representative staining. Kidney tissue served as a positive control. **D)** Percent ERRα staining on the tissue microarray is shown. Data represent mean ± SEM of three independent experiments in A and B. Statistical significance assessed with Kruskal-Wallis test with Bonferroni correction for multiple comparisons.

In contrast to the murine *Kit*^*ΔV558/+*^ model, human GIST cell lines (T1, HG129, and HG130) and 6 surgical tumor specimens (HG1–6) had minimal ERα mRNA and protein expression (Fig. 2A), consistent with previous reports that GISTs do not express ERα (29, 30). Given that ERα was clearly driving tumor biology in the murine model but was absent from human tumors, we reasoned that a related nuclear receptor might be involved in human GIST. Therefore, we quantified the relative RNA expression of estrogen receptor-β (*ESR2*), estrogen-related receptors-α,-β,and -γ (*ESRRA, ESRRB, ESRRG*), and progesterone receptor (*PGR*) in 2 human GIST cell lines (T1, 882) and 75 human GIST tumor specimens using a publicly available dataset and our prior published data, respectively (26, 27). ERRα was expressed at dramatically higher levels than the other nuclear receptors in the cell lines and tumor specimens (**Supp.** Figure 2). To validate this finding, we assessed ERRα mRNA and protein expression in the same human GIST cell lines and surgical tumor specimens from Fig. 2A, which confirmed robust ERRα expression across most cell lines and tumor samples (Fig. 2B). Additionally, a tissue microarray of 66 untreated primary GISTs showed staining in most samples, although the extent varied (Fig. 2C–D). Staining mostly localized to the cytoplasm, which is consistent with other reports despite ERRα being a nuclear receptor and transcription factor (31, 32). Expression did not depend on tumor mitotic rate, location, or gender (**Supp.** Figure 3). Further analysis of our published RNA-seq data (27) did not show a difference based on treatment status or mutation (**Supp.** Figure 4).

### ERRα inhibition suppresses GIST cell proliferation in vitro and in vivo.

**Figure 3:**
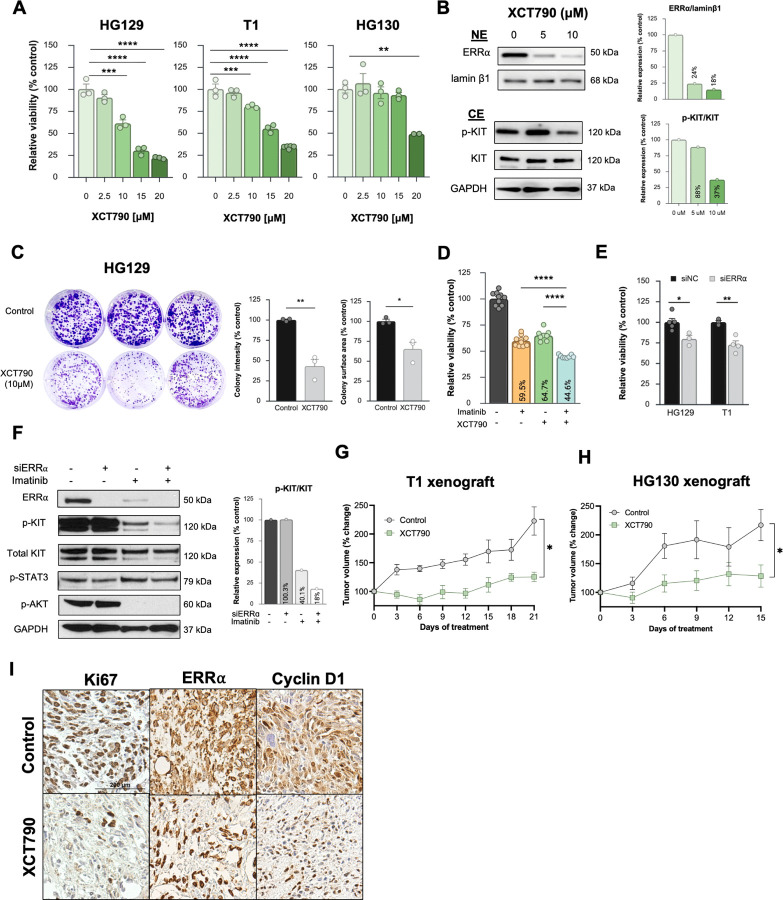
ERRα inhibition suppresses GIST cell proliferation *in vitro* and *in vivo*. Relative cell viability compared to control of: **A)** HG129, T1, and HG130 GIST cell lines treated with XCT790 for 72h. **B)** Western blot of HG129 cells after 72h treatment with XCT790 with densitometric quantification of ERRα (top, normalized to lamin-b1) and p-KIT (bottom, normalized to total KIT).**C)** Representative images of colony formation (left) and quantification of staining intensity and surface area (right) in HG129 cells treated with 10 μM XCT790 or vehicle for 48h. **D)**Relative viability of T1 cells treated with imatinib (25 nM), XCT790 (10 μM), or both for 72h. **E)**Relative viability of HG129 and T1 cells after transfection with ERRα siRNA (siERRα) or non-target control siRNA (siNC) at 48h. **F)** Western blot of HG129 cells treated with siERRα, imatinib, or both with densitometric quantification of p-KIT/total KIT. **G)**T1 xenografts were randomized to daily XCT790 5 mg/kg (n=5) or vehicle (n=4). **H)** HG130 xenografts were randomized to daily XCT790 10 mg/kg or vehicle (5 mice/group). **I)** Representative immunohistochemistry of T1 xenografts from G. Data represent mean ± SEM. Statistical significance was assessed by one-way ANOVA with Tukey’s post-hoc test (A, C) and unpaired Student’s t-test (B, F, G) *p<0.05, **p<0.01, ***p<0.001, ****p<0.0001.

To determine if ERRα contributes to GIST progression, 3 human GIST cell lines (T1, HG129, HG130) were treated with XCT790, an ERRα inverse agonist (33), which decreased proliferation in a dose-dependent manner (Fig. 3A). At 72h, the IC_50_ ranged from 10.8 to 19.0 μM (**Supp.** Figure 5), consistent with doses used in other cancer cell lines (34, 35). In HG129 cells, XCT790 reduced nuclear ERRα, verifying its targeting effects (Fig. 3B). Notably, cytoplasmic phospho-KIT protein was also reduced (Fig. 3B), as was colony intensity and surface area compared to control (Fig. 3C). Combining imatinib with XCT790 further reduced T1 cell viability beyond either agent alone (Fig. 3D) To prove specificity, we performed siRNA-mediated ERRα knockdown in T1 and HG129 cells. ERRα knockdown suppressed cell viability after 72h (Fig. 3E). When combined with imatinib in HG129 cells, ERRα knockdown further reduced phospho-KIT levels, but not phospho-STAT3, or phospho-AKT (Fig. 3F). Additionally, ERRα knockdown at 48h did not suppress mRNA expression of *KIT* or the transcription factors *ETV1* and *FOXF1* (**Supp. Figure 6**), which enhance KIT expression and are essential for GIST growth (36, 37).

To determine the effect of XCT790 on tumor growth *in vivo*, established T1 and HG130 xenografts were treated with daily gavage of XCT790 or vehicle for 3 weeks. XCT790 markedly reduced tumor growth in both models without causing appreciable toxicity (Fig. 3G–H). Immunohistochemistry of T1 xenografts confirmed that XCT790 therapy reduced tumor Ki-67, ERRα, and cyclin D1 expression (Fig. 3I).

### ERRα regulates EMT in GIST.

**Figure 4:**
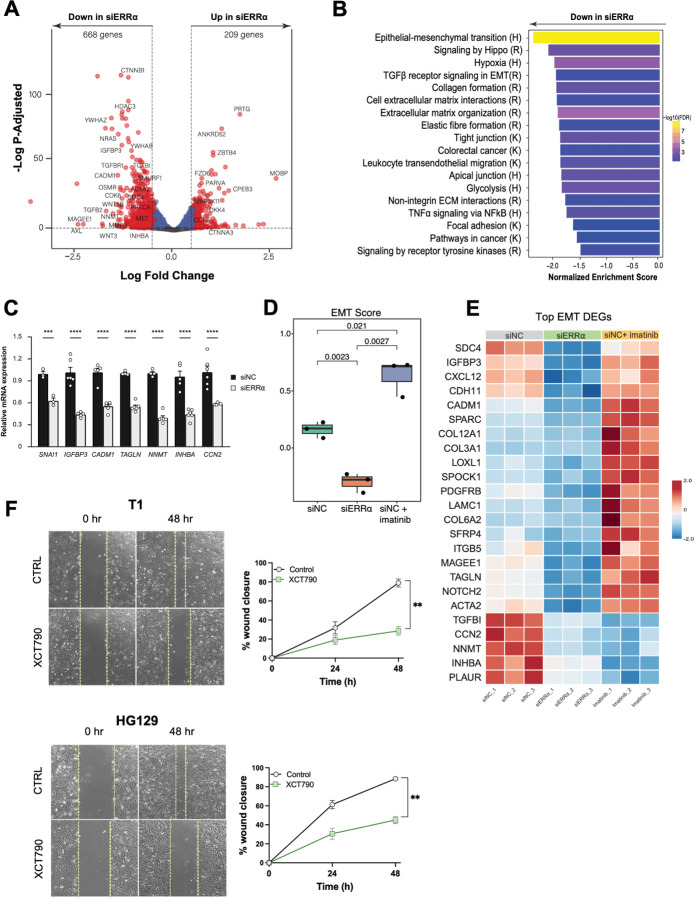
ERRα knockdown suppresses EMT. **A)**Volcano plot of differentially expressed genes (DEGs) after siRNA-mediated ERRα knockdown (siERRα) in GIST T1 cells. Dashed lines indicate log2FC = ±0.5 and padj <0.05 thresholds. Red is significant and blue is non-significant. **B)**Gene set enrichment analysis (GSEA) bar plot showing selected significantly downregulated gene sets (Hallmark, KEGG, Reactome) following ERRα knockdown, ranked by Normalized Enrichment Score (NES). **C)** Validation of RNA-seq data by qPCR of selected EMT DEGs in T1 cells after siERRα vs. knockdown of not-target control (siNC, n=3/group). **D)** EMT signature score in T1 cells following siERRα knockdown and imatinib treatment compared to siNC. **E)**Heatmap of leading-edge EMT genes from the Hallmark EMT gene set following siERRα knockdown. Color scale represents log2 fold change; Z-score normalized per gene. **F)** Representative images (left, 4x magnification) and quantification (right) from a scratch assay in T1 and HG129 cells treated with XCT790 5 mM or vehicle (n=3/group). Data represent mean ± SEM of 3 independent experiments for C and F. Statistical significance assessed with Two-way ANOVA with Bonferroni multiple comparisons test (C), Welch’s one-way ANOVA (D), and Unpaired t-test (F). *p<0.05, **p<0.01, ***p<0.001, ****p<0.0001.

To investigate the role of ERRα in regulating GIST gene expression, we performed bulk RNA sequencing of T1 cells after siRNA-mediated ERRα knockdown. Principal component analysis (PCA) demonstrated clear separation versus non-target control (**Supp. Figure 7**). There were 991 differentially expressed genes (DEGs), 668 downregulated and 209 upregulated (Fig. 4A). Notable downregulated genes were *CTNNB1* (β-catenin), *IGFBP3, TGFBI, TGFBR1, CADM1, AXL, IGFBP3*, and *CDK6*, while upregulated genes included *FZD6, DKK4* and *ZBTB4*. Gene Set Enrichment Analysis (GSEA) (21) revealed the top downregulated pathway to be Hallmark Epithelial Mesenchymal Transition (EMT) (Fig. 4B). Numerous other downregulated pathways spanned EMT and cell migration, including TGFβ Receptor Signaling in EMT, Leukocyte Transendothelial Migration, and Focal Adhesion; extracellular matrix remodeling gene sets, including Collagen Formation, Cell Extracellular Matrix Interactions, and Extracellular Matrix Organization; cell junction programs, including Tight Junction and Apical Junction; as well as others (**Supp. Figure 8)**.

We confirmed the RNA-seq findings using quantitative PCR, which showed that ERRα knockdown downregulated numerous selected EMT DEGs (Fig. 4C). EMT score (25), which is based on the Hallmark EMT gene set, was decreased by ERRα knockdown compared to non-target control (Fig. 4D). Surprisingly, though, EMT was increased by imatinib (Fig. 4E, **Supp. Figure 9**). Imatinib upregulated 115/199 genes from the Hallmark gene set (**Supp. Table 3**), including *IGFBP3*, *CDH11*, *NOTCH2*, and *PDGFRB*, which have all been found to promote progression and metastasis in various types of sarcoma (38–41). To functionally validate the effect of ERRα knockdown on EMT, we assessed cell migration via scratch assay and found that XCT790 reduced wound closure in both T1 and HG129 cells (Fig. 4F).

### ERRα promotes EMT via β-Catenin.

**Figure 5:**
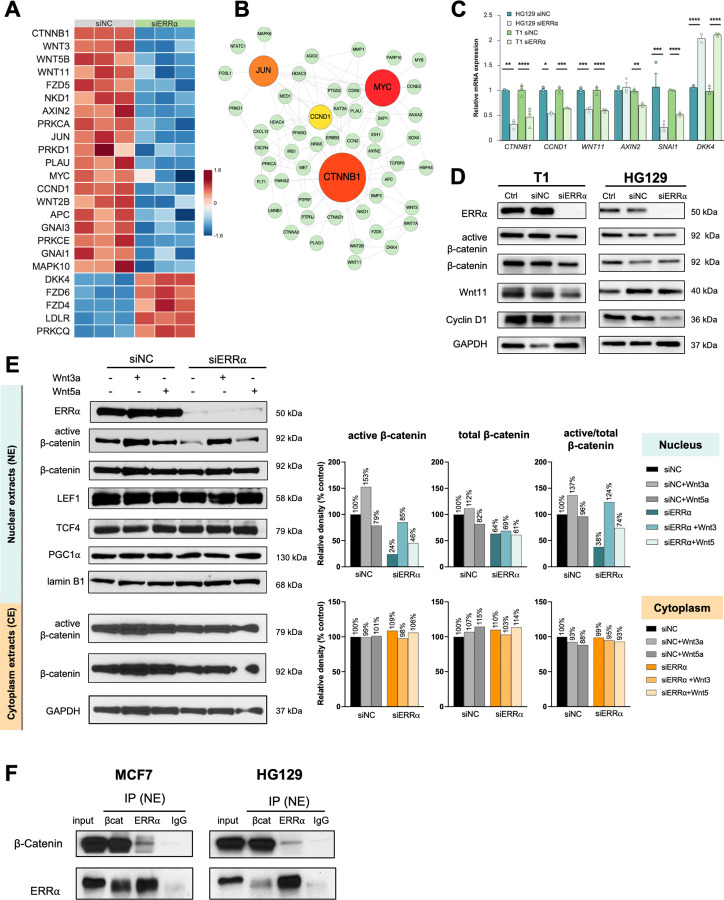
ERRα promotes EMT via regulation of Wnt/β-catenin in GIST. A) Heatmap of alterations of DEGs involved in Wnt/β-catenin signaling and gene targets following knockdown of. ERRα (siERRα) or non-target control (siNC). Color scale represents log2 fold change; Z-score normalized per gene. **B)**STRING protein-protein interaction (PPI) network of DEGs following ERRα knockdown. Node size reflects degree of interaction; β-catenin (*CTNNB1*) is highlighted as the central hub gene. **C)**qPCR validation of β-catenin (*CTNNB1*) and target gene expression in T1 and HG129 cells following ERRα knockdown (siERRα) compared to non-target control (siNC). **D)** Western blot analysis following ERRα knockdown compared to non-target and control (DMSO) groups in T1 and HG129 cells. **E)** Nuclear-cytoplasmic fractionation of T1 cells treated with siERRα or siNC, with or without Wnt3a or Wnt5a (100 ng/mL). Lamin B1 was used as a loading control in the nuclear fraction. **F)** Western blot of nuclear extracts (NE) from untreated HG129 and MCF7 cells alone (input) or after immunoprecipitation of β-catenin, ERRα, and IgG control. Data represent mean ± SEM of 3 independent experiments unless otherwise noted. Statistical significance assessed with Two-way ANOVA with Bonferroni multiple comparisons test. *p<0.05, **p<0.01, ***p<0.001.

To determine the mechanism by which ERRα drives EMT, we considered the numerous pathways known to induce EMT, including Wnt/β-catenin, TGFβ, and Notch, among others. Since we previously found that inhibition of Wnt/β-catenin signaling suppressed tumor growth in preclinical GIST models, we focused on this pathway (11, 13). Using our RNA-seq data from ERRα knockdown, we identified that in addition to *CTNNB1*, numerous other genes involved in Wnt signaling (*WNT11, FZD5*, and *AXIN2*) as well as Wnt/β-catenin target genes (*MYC* and *CCND1* (cyclin D1)) were downregulated (Fig. 5A). Protein-protein interaction (PPI) network analysis using our DEGs reveled β-catenin (*CTNNB1*) as the top hub gene (Fig. 5B). qPCR analysis confirmed lower *CCND1, WNT11, AXIN2, and SNAI1* mRNA expression in both T1 and HG129 cells after ERRα knockdown (Fig. 5C). Notably, *DKK4*, a Wnt target gene that antagonizes Wnt signaling in a negative feedback loop, was markedly upregulated after knockdown (Fig. 5C).

To determine whether ERRα knockdown affects total β-catenin protein, we performed western blots on whole cell lysates from T1 and HG129 cells. Total and active (non-phosphorylated) β-catenin were reduced, as were the downstream targets Wnt11 and Cyclin D1 (Fig. 5D). To isolate which cellular compartment was affected by ERRα knockdown, we fractionated cytoplasmic and nuclear extracts after ERRα knockdown with or without stimulation of canonical (Wnt3a) or non-canonical (Wnt5a) Wnt ligands. ERRα knockdown alone reduced active and total β-catenin in the nucleus while cytoplasmic levels were relatively unaffected (Fig. 5E). Wnt3a stimulation, and to a lesser extent Wnt5a, partially rescued nuclear active β-catenin. ERRα knockdown did not alter the levels of the nuclear β-catenin cofactors LEF1 and TCF4 and the ERRα coactivator PGC1α. To determine if ERRα and β-catenin interact at the protein level, we performed co-immunoprecipitation assays of nuclear extracts from HG129 cells. MCF7 cells were used as a positive control since ERRα–β-catenin binding has been shown in breast cancer (42). Indeed, in MCF7 and HG129 cell lines, a β-catenin antibody co-precipitated ERRα at 50–55 kDa, and conversely an anti-ERRα antibody co-precipitated β-catenin at 92 kDa (Fig. 5F).

## DISCUSSION

Imatinib has dramatically transformed the prognosis of advanced GIST, yet it is rarely curative. While most patients show an initial tumor response, secondary resistance commonly ensues. Secondary KIT mutations are the most common mechanism of acquired resistance, but evidence suggests these permanent genetic alterations tend to arise in the late stages of resistance (43). Early resistance is driven by reversible, non-genetic adaptations that confer a survival benefit to a subpopulation of tumor cells, allowing them to persist during imatinib treatment. In this study, we identified ERRα, albeit in a circuitous manner, as a novel oncogenic driver in GIST that promotes tumor growth and EMT through regulation of nuclear β-catenin. These findings provide a rationale for targeting the ERRα-β-catenin axis as a therapeutic strategy in GIST, particularly in combination with standard TKI therapy to reduce the development of resistance.

ERRα regulated both an EMT transcriptional program and phenotype in GIST models, consistent with its oncogenic role in other cancers (35, 42, 44, 45). Our RNA-seq data revealed that EMT was the most significantly downregulated gene set following ERRα knockdown. ERRα knockdown also suppressed multiple EMT-inducing pathways, including β-catenin, TGF-β and Hippo, which do not share overlapping leading-edge genes, signifying that ERRα operates as a systems-level upstream regulator of EMT rather than through a single-pathway effector. ERRα knockdown also suppressed gene sets distinct from the core EMT signature that involve cell junction integrity and migration, which represent additional programs relevant to regulation of invasion and metastasis. This breadth of the transcriptional impact of ERRα has important therapeutic implications, since targeting individual EMT transcription factors and EMT-inducing pathways has historically proven insufficient due to compensatory cross-talk and parallel signaling (7). Notably, EMT was the most significantly enriched pathway in liver metastases compared to primary GIST tumors in a recent transcriptomic study, further supporting the clinical relevance of ERRα-driven EMT as a driver of disease progression (46).

GIST response to imatinib culminates in a proportion of persistent cells that survive in a dormant state, but can resume proliferation once treatment is discontinued (3). It has been theorized that this drugtolerant state can eventually contribute to genetically-driven resistance, but the mechanisms that enable initial persistence are unclear (3, 43). Our results indicate that imatinib has a net stimulatory effect on EMT genes, suggesting a possible mechanism that facilitates tumor cell persistence. While short-term imatinib therapy upregulated EMT in the cell lines despite reducing the amount of ERRα protein, it is notable that ERRα expression did not differ among human surgical specimens that prior to surgery were untreated, or treated generally for at least 6 months and remained responsive or had become resistant to imatinib. Nevertheless, it is likely that there are other ERRα-independent mechanisms to increase EMT. Although ERRα knockdown alone did not suppress KIT, ETV1, or FOXF1 mRNA, the combination of imatinib with either ERRα knockdown or XCT790 reduced phospho-KIT beyond what imatinib achieved alone. Combined with the finding that XCT790 augments imatinib’s effects on cell line viability, these results suggest that ERRα inhibition and KIT-targeted therapy are complementary rather than redundant.

Our previous work established Wnt/β-catenin signaling as an independent driver of GIST aggressiveness and demonstrated that combining a Wnt/β-catenin inhibitor with imatinib provided greater tumor suppression and KIT signaling inhibition than either agent alone (11, 13). In the current study, β-catenin (*CTNNB1*) emerged as the top hub gene in our protein-protein interaction (PPI) network analysis of ERRα-regulated genes, and subsequent experiments confirmed that ERRα knockdown reduces nuclear active β-catenin, and downstream Wnt target genes including Cyclin D1, Wnt11, and Snai1. In prostate and breast cancer cell lines, *WNT11* was identified as a shared transcriptional target of ERRα and β-catenin and found to promote cell migration (42). We also found that *DKK4* levels increased dramatically after knockdown, which our previous work identified as a negative regulator of Wnt signaling in GIST, effectively reducing active β-catenin, p-GSK3β, and cyclin D1 (13). Like *AXIN2*, *DKK4* is a target gene normally upregulated by Wnt/β-catenin signaling as part of a negative feedback loop (47). However, unlike most downstream target genes that showed decreased expression after ERRα knockdown, *DKK4* expression increased substantially despite β-catenin inhibition. This suggests ERRα may regulate *DKK4* through a different mechanism independent of β-catenin activity. One possibility is that ERRα normally represses DKK4 transcription, and knockdown results in acute release of this brake and a spike in DKK4 expression before compensatory mechanisms develop. In neuronal cells, ERRα was found to directly repress DKK1 expression, which prevented negative feedback on Wnt signaling and maintained pathway activation (48). Interestingly, the same study also found that ERRα directly binds the *CTNNB1* promoter region but did not detect significant change in mRNA expression after ERRα depletion. Mechanistically, our results indicate that ERRα is important for maintaining levels of transcriptionally active nuclear β-catenin and that they physically interact at a protein level. Further investigation is expected to delineate whether ERRα directly promotes *CTNNB1* or represses *DKK4* at a transcriptional level.

ERRα represents a druggable target, and XCT790 has demonstrated anti-EMT efficacy across cancer types by suppressing migration, invasion, and metastatic spread (35, 44, 49). While XCT790 has limitations for clinical development, next-generation ERRα inverse agonists are under active investigation (50). Importantly, XCT790 suppressed cell viability in HG130, an imatinib- and sunitinib-resistant cell line, demonstrating that ERRα inhibition retains activity in the TKI-resistant setting where therapeutic options are most limited. ERRα also emerged as an important β-catenin regulator, which historically has been difficult to target because it lacks enzymatic activity. This makes upstream regulators like ERRα attractive entry points into the Wnt/EMT axis, complementing ongoing efforts with porcupine inhibitors and β-catenin/TCF blockers that are currently in clinical trials (51). Taken together, our findings suggest that an ERRα inhibitor with imatinib may be a complementary therapeutic combination to suppress unwanted EMT activation and potentially reduce tumor cell survival and persistence, which often culminates in resistance.

## Supplementary Material

Supplementary Files

This is a list of supplementary files associated with this preprint. Click to download.

- SupplementaryTables13.xlsx
- SupplementaryTable2.xlsx
- SupplementaryTable3.xlsx
- SupplementaryFigures.pdf

## Data Availability

RNA-sequencing data have been deposited in NCBI’s Gene Expression Omnibus (GEO) and are accessible through GEO Series accession number GSE330412. Publicly obtained data analyzed in this study were obtained from GEO at GSE143547 and Sequencing Read Archive at accession PRJNA521803. All remaining data supporting the findings of this study are available within the article and its supplementary materials or from the corresponding author upon reasonable request.
